# Cellulose-based sensors for decentralized monitoring in precision agriculture

**DOI:** 10.1038/s41467-026-70730-7

**Published:** 2026-03-13

**Authors:** Mirinal K. Rayappa, José M. R. Flauzino, Max Grell, Firat Güder

**Affiliations:** 1 Department of Bioengineering, Royal School of Mines, Imperial College London, London, UK; 2 BlakBear Ltd, London, UK; 3 Bezos Centre for Sustainable Protein, Imperial College London, London, UK

**Keywords:** Biochemistry, Electrochemistry, Plant sciences, Sensors and probes

## Abstract

Modern agriculture requires rapid, affordable tools to monitor crops and soils directly in the field. Cellulose, the structural polymer of plants, is emerging as a versatile foundation for lightweight, biodegradable sensors that measure nutrients, moisture, stress, and disease without laboratory infrastructure. Spanning simple paper assays to flexible wearables, these platforms enable distributed, real-time insight into plant and soil health. As materials engineering converges with digital connectivity, cellulose-based sensors could accelerate the transition toward more data-driven, adaptive, and sustainable agricultural systems.

## Abstract (web summary)

Agriculture is turning to precision application of agrochemicals and growth conditions, but to achieve this affordable tool to monitor crops and soils in field are needed. Here, the authors review the use of cellulose-based materials to make flexible wearable sensors that allow for in situ monitoring of agricultural conditions.

## Introduction

Despite the productivity gains of the Green Revolution, modern agriculture remains fundamentally inefficient: only ~6% of global biomass is converted into consumable food, despite the sector employing nearly half of the world’s workforce^1^. Intensive practices such as deep tillage, excessive agrochemical use, and mismanaged irrigation have accelerated soil degradation worldwide: by 2015, an estimated 33% of global soils were moderately to highly degraded, with over half of agricultural land affected by erosion, salinization, acidification, contamination, or compaction^2–4^. This deterioration carries profound economic and food-security consequences, costing US$400 billion annually and threatening further declines in productivity and rising food prices^5^. Coupled with shrinking arable land, freshwater scarcity, climate change, and increasing vulnerability to stress and disease, these pressures now challenge the long-term resilience of global food systems^6,7^.

To overcome these challenges, technological innovation in agriculture is essential. Precision agriculture, which involves the adoption of intelligent technologies, such as sensors, information systems, and communication networks, enables data-driven, site-specific actions to optimize agricultural production and monitor activities in real time by capturing the spatio-temporal dynamics of numerous soil and plant conditions^8,9^. Integral to these activities are point-of-use sensors, which serve as essential tools in precision agriculture by enabling these real-time data collection without the need for centralized laboratories or staffed operations. These sensors offer key advantages, including low cost, portability, rapid diagnostics, minimal storage requirements, and suitability for use in resource-limited settings. They typically incorporate biological, chemical, or physical sensing mechanisms within miniaturized or reusable devices, allowing point-of-use analysis^10^.

**Fig. 1:**
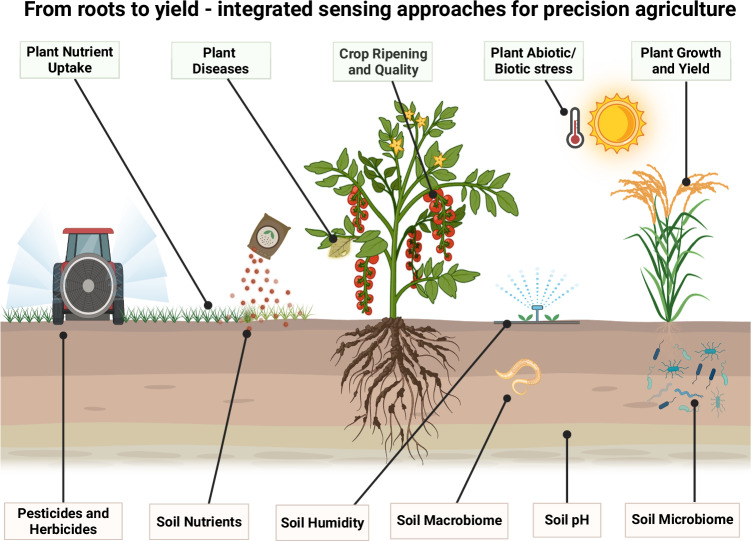
Point-of-use sensing approaches for precision agriculture. Schematic overview of on-site analytical strategies used in farming environments to monitor plant and soil health. These approaches collectively enable precision agriculture, in which real-time data acquisition supports optimized crop management and sustainable productivity. Key plant-related parameters include nutrient uptake, disease occurrence, crop ripening and quality, responses to abiotic and biotic stress, and overall growth and yield. Belowground sensing focuses on soil-related indicators such as pesticide and herbicide residues, nutrient composition (N, P, K, Ca), moisture content, macrobiome and microbiome diversity, and pH variations. Integrated point-of-use platforms, ranging from colourimetric strips and electrochemical probes to wireless biosensors, provide rapid, low-cost, and field-deployable diagnostics that inform irrigation, fertilization, and crop protection decisions while reducing environmental impact. Created with BioRender.com.

In this context, it is important to distinguish between *point-of-use* and *decentralized* sensing. Point-of-use sensing refers to analytical measurements performed directly at the sampling location, removing reliance on centralized laboratories, whereas decentralized sensing builds on this concept by enabling measurements to be acquired across multiple spatial locations over meaningful time scale which are then digitally networked and integrated via data-driven analytics to support real-time, site-specific decision making Accordingly, while many sensing devices do operate at the point of use, their greatest impact in precision agriculture emerges when they are deployed as part of decentralized sensing networks^11,12^. Originally developed for healthcare, point-of-use sensing technologies are now increasingly applied across agriculture and food systems, supporting resource monitoring, agrochemical management, crop and soil health assessment, and pre- and post-harvest quality control (Fig. 1).

Several key factors have driven the increasing adoption of point-of-use sensing in agriculture. A major contributor is the progress in molecular sensor technologies, which, when integrated with digital platforms, allows for the detection of a broad range of analytes such as heavy metals, agrochemicals, nutrients, biomarkers, and pathogens in soil and plant systems^13–16^. Second, declining costs and improved performance of opto-electronic components are enabling more accessible sensing platforms: low-cost optical, electronic, and microelectromechanical devices now support compact, integrated measurements outside laboratory settings. At the same time, Internet-of-Things connectivity, including near-field communication (NFC), Bluetooth, and 5G, enables wireless data transmission and distributed deployment of sensors at scale. When combined with cloud computing and artificial-intelligence-based data analysis, these technologies allow efficient data collection, interpretation, and decision-making, even in remote or resource-limited environments^17,18^. Third, rising labor costs and the increasing automation of agricultural operations demand more data-driven decision-making. Finally, the urgent need to monitor and reduce the environmental impact of farming has brought renewed attention to sustainable materials.

As these technological, economic, and environmental factors shape the future of point-of-use sensing in agriculture, they also underscore the need for substrates that are not only compatible with low-cost, digitally integrated platforms but also sustainable and field-ready. This convergence of performance and environmental responsibility has thus directed growing attention toward naturally derived materials that can support scalable, disposable, or biodegradable sensing formats. Among these, cellulose stands out as a particularly compelling option, offering a uniquely favorable combination of abundance, mechanical robustness, chemical tunability, and environmental compatibility.

## Cellulose as a sensing material

**Fig. 2:**
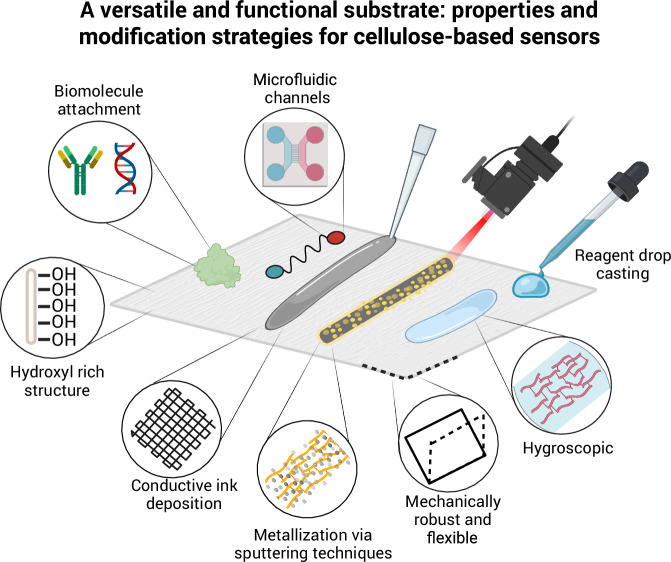
Common surface functionalization and fabrication methods used for developing analytical sensors with cellulose fabrics as substrates. The inherent porosity, hydroxyl-rich surface chemistry, and fibrous morphology of cellulose enable diverse functionalization strategies, including covalent grafting, adsorption, silanization, polymer coating, nanoparticle immobilization, and inkjet or screen printing. These modifications support the attachment of biorecognition elements such as antibodies, enzymes, nucleic acids, or aptamers, as well as the incorporation of nanomaterials (e.g., metal nanoparticles, graphene inks), polymers (e.g., polydiacetylenes), and signal labels (e.g., dyes, fluorophores, redox probes). Together, these features enable the integration of colourimetric, fluorescent, Raman, and electrochemical modalities onto flexible and biodegradable platforms. Created with BioRender.com.

Cellulose is the most abundant organic polymer on Earth, serving as the primary structural component of plants^19^. Composed of repeating β-D-glucose units linked by β−1,4-glycosidic bonds, it forms robust, hydrogen-bonded networks that provide mechanical strength and stability. Cellulose is renewable, biodegradable, and chemically versatile, making it a valuable material across diverse fields. Its hydroxyl-rich surface allows for versatile biochemical molecule functionalization and surface engineering (e.g., oxidation of -OH groups into -COOH, which increases surface hydrophilicity and cellulose-metal coordination chemistry)^20^, enabling advanced applications in textiles, packaging, medicine, and sensing (Fig. 2)^21^.

Beyond serving as a passive support, cellulose increasingly functions as an active sensing material whose surface chemistry, porosity, dielectric behavior, and chemical modifiability directly influence analyte capture, signal transduction, and analytical sensitivity. Cellulose’s continued adoption across diverse sensing platforms reflects a convergence of material properties that are rarely unified in a single substrate. Its porous, fibrous architecture simultaneously enables passive fluid transport, reagent storage, and high interfacial surface area, while its hydroxyl-rich surface chemistry supports covalent and non-covalent functionalization with biological recognition elements, nanomaterials, and redox labels. At the device level, cellulose combines mechanical flexibility, optical transparency or opacity (depending on processing), and tunable dielectric behavior, allowing seamless integration with colourimetric, fluorescent, electrochemical, and electrical transduction strategies^22^.

Unlike conventional polymeric or silicon-based substrates, cellulose supports low-temperature, additive manufacturing and roll-to-roll processing while remaining biodegradable and environmentally benign. These attributes make cellulose not merely a passive support, but an active materials platform capable of coupling fluid handling, chemical recognition, and signal transduction within scalable, field-deployable sensor architectures^23^. As a result, cellulose functions as a unifying substrate across point-of-use, wearable, and wireless sensing modalities relevant to precision agriculture.

As summarized in Table 1, cellulose can be processed into a wide range of substrates, from inexpensive paper and textile fibers to engineered bacterial cellulose, each offering distinct trade-offs in cost, structure, and functional performance for applications in sensing. Paper is the most widely used cellulose-based material due to its low cost and ease of processing. Other cellulose fabrics, such as cotton, lyocell, viscose, nitrocellulose, and bacterial cellulose, offer unique advantages for specific sensing applications. For instance, bacterial cellulose provides thermal stability, high purity, and tunable properties, making it ideal for customizable sensor designs^24^. Nanocellulose, including cellulose nanofibrils and cellulose nanocrystals, introduces an additional class of substrates with high specific surface area porous networks, optical transparency, and tunable nanoscale ordering, enabling mechanically robust films and intrinsic optical sensing architectures without the need for additional functional fillers^25^. Viscose and lyocell belong to the broader family of regenerated cellulose fibers, where materials are produced by dissolving natural cellulose (typically from wood pulp) and re-forming it into highly uniform fibers through controlled chemical regeneration. This processing step disrupts the native hierarchical structure of cellulose and rebuilds it with tunable crystallinity, porosity, and mechanical behavior, resulting in substrates with far more consistent physical properties than raw paper. Viscose, for example, provides high tensile strength and good dimensional stability, while lyocell offers exceptionally uniform fiber distribution and enhanced moisture-handling capacity, features that are particularly valuable for environmental sensing applications where controlled wicking and predictable analyte transport are critical^26,27^. Nitrocellulose is a cellulose-derived polymer produced by treating cellulose with nitric and sulfuric acids, resulting in a highly porous, hydrophilic material with protein-binding properties. It is extensively used for lateral flow assays (LFAs) assembling^28^.

**Table 1:** Comparative properties of cellulose-based substrates used in sensor fabrication

Type of cellulose	Composition/Source	Approx. Cost (USD/m²)*	Advantages for sensing	Disadvantages/Limitations
Paper (Whatman, filter, chromatographic)	Plant-derived cellulose fibers (β-D-glucose, β−1,4 linkages)	5–15	Low cost; easy patterning and cutting; excellent capillary action; compatible with colourimetric and electrochemical assays	Non-uniform fiber structure; low wet strength; humidity-dependent performance
Cellulose acetate	Acetylated cellulose with tunable degree of substitution; castable films and/ or membranes	40–80	Smooth, uniform films; tunable porosity and permeability; good mechanical stability; useful as analyte‑permeable protective/encapsulation layer; low‑cost membrane processing (casting, spin/doctor‑blade)	Reduced surface reactivity (fewer –OH groups); requires activation; often hydrophobic; solvent processing uses acetone/DMF; slower capillary flow than paper
Cotton fabric	Natural cellulose fibers with minor waxes/lignin	20–40	Flexible, breathable, reusable; good mechanical durability; biocompatible for wearable devices	Surface roughness and impurities require pretreatment; variable wicking rate
Lyocell	Regenerated cellulose dissolved in N-methylmorpholine N-oxide (NMMO)	30–60	Uniform fiber morphology; excellent moisture management; consistent electrical/mechanical behavior	Higher cost; less chemically active surface than native cellulose
Viscose (Rayon)	Regenerated cellulose via xanthate process	25–50	High tensile strength; smooth surface for uniform coating; scalable textile format	Environmental concerns from viscose production: lower porosity than paper
Nitrocellulose	Cellulose nitrate (nitrated with HNO₃/H₂SO₄)	200–500	High protein-binding capacity; ideal for lateral flow assays (LFA) and biofunctionalization	Flammable; limited mechanical stability under high humidity; expensive
Bacterial cellulose/Nanocellulose	Microbially synthesized (e.g., *Komagataeibacter xylinus*)	300–800	High purity and crystallinity; tunable porosity/thickness; excellent flexibility and water retention; ideal for biosensing membranes	High production cost; slower scale-up; sensitive to dehydration

^*^Approximate costs were based on current market prices (Sigma-Aldrich, ThermoFisher Scientific, VWR, and Cole-Parmer catalogs, accessed November 2025) for laboratory-grade, high-purity cellulose materials typically used in sensor fabrication. Reported values represent the average price per square meter calculated from available sheet, membrane, or roll formats. These costs are higher than those of industrial-grade cellulose due to enhanced purity, uniformity, and certification standards required for analytical and biosensing applications.

**Fig. 3:**
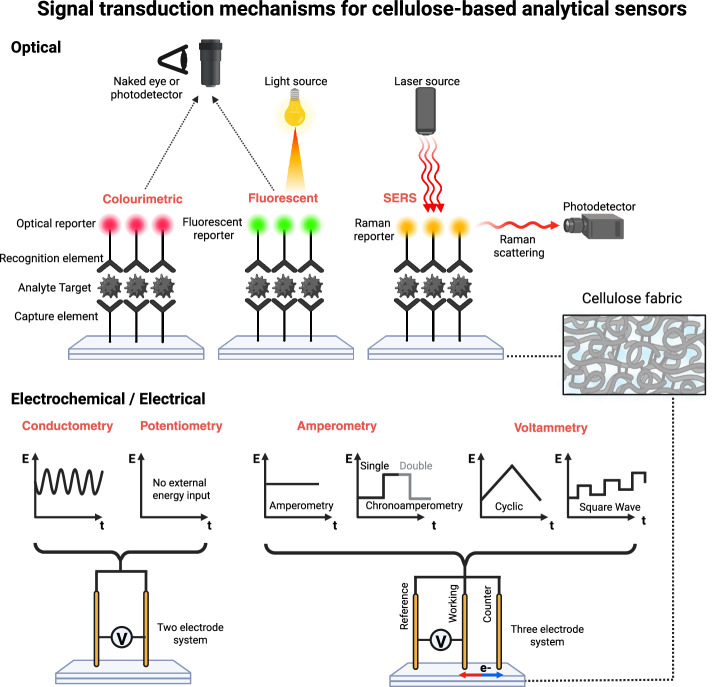
Transduction strategies for cellulose-based analytical sensors. Optical readouts rely on colourimetric or fluorescent responses detectable by the naked eye or optical readers, while electrochemical detection, using conductometric, potentiometric, amperometric, or voltametric techniques, enables quantitative and multiplexed signal acquisition. The combination of porous cellulose matrices with miniaturized electrodes facilitates sensitive and low-cost biosensing suitable for wearable, point-of-care, and environmental monitoring applications. Created with BioRender.com.

Figure 3 illustrates the primary transduction mechanisms employed in cellulose-based sensing platforms, highlighting both optical and electrochemical approaches. These methods translate molecular recognition events into measurable optical or electrical outputs, enabling rapid and decentralized analysis. Optical readouts, including colourimetric, fluorescent, and Raman-based modes, provide visually intuitive signals while also supporting quantitative and multiplexed analysis. Electrochemical detection similarly enables quantitative and multiplexed measurements through current, potential, or impedance variations.

Colourimetric devices based on cellulose fabrics offer a distinct advantage in that they can be operated by users with minimal technical expertise, as the readout is made by the naked eye. They typically employ pH-sensitive dyes, metal nanoparticles, color-changing enzymatic substrates, supramolecules, and polymers (e.g., polydiacetylene), which produce analyte-specific chromatic shifts through biomolecule recognition, dye interactions, and/or nanoparticle aggregation^29–31^. Besides qualitative results, colourimetric devices can enable quantitative interpretation, as data can be recorded directly at the point of use using a smartphone camera or a photodetector. Digital image analysis tools such as ImageJ, Ilastik, and Icy can quantify colourimetric signals using models like RGB, HSV, or CIELAB^32^. This quantification enables indirect but reliable correlation of color with analyte concentration, useful for portable molecular diagnostics that require minimal or no instrumentation for readout.

Instrumented optical modalities represent a distinct class of cellulose-based sensors that rely on externally driven photonic interrogation rather than visual inspection. Fluorescent platforms illustrate one end of this spectrum where molecular or nanoscale emitters (e.g., organic dyes, lanthanide chelates, quantum dots, etc.) are embedded within cellulose fibers to form chemically responsive microenvironments. Analyte recognition modulates their photophysical behavior through static or dynamic quenching, resonance energy transfer, or emission enhancement, often supporting detection limits in the nano- to picomolar range. Achieving such performance requires controlled optical excitation and emission capture, typically via LED or laser sources coupled to photodiodes or compact spectrometers^33^. Building on the same instrumented principles, Raman spectroscopy captures molecular vibrational fingerprints rather than relying on emission-based signals. Surface-enhanced Raman spectroscopy (SERS) on cellulose can deliver orders-of-magnitude signal amplification when the substrate incorporates gold or silver nanoparticle clusters (10-50 nm) with nanogap distributions below 3–5 nm, generating well-defined plasmonic hot spots^34^. Enhancement factors of 10⁶−10⁸ are routinely reported under these conditions, enabling picomolar–femtomolar quantification and multiplexed molecular profiling with handheld 532–785 nm Raman readers. Performance nonetheless remains tightly coupled to fabrication quality, where insufficient nanostructural control leads to batch-to-batch variability exceeding 30–50%, and long-term stability is still limited by humidity, oxidation, and mechanical deformation. Together, fluorescence and SERS define the high-sensitivity, instrumentation-dependent arm of cellulose optical sensing, offering spectral richness and ultralow detection limits that complement the simplicity of naked-eye assays while imposing stricter requirements on excitation hardware, substrate uniformity, and environmental robustness^35^.

Electrochemical and electrical transducers convert chemical interactions between an analyte and a recognition element into quantifiable electrical signals. These systems rely on redox or ionic reactions occurring at the surface of an electrode, which is often fabricated by integrating conductive inks, such as carbon, silver, or gold, onto cellulose-based substrates^36^. Patterned conductive surfaces can be produced using methods like plating, sputtering, 3D printing, or laser scribing, which create defined electroactive regions on the fabric. These transducers typically operate via amperometric, potentiometric, or impedance-based mechanisms, enabling sensitive and selective detection of target analytes^37,38^. Recent progress in cellulose-based electrochemical sensors has been focused on improving charge transfer efficiency, environmental stability, and multi-analyte detection capability. Incorporating nanostructured materials such as graphene, MXenes, metal–organic frameworks, and carbon nanotubes into cellulose matrices enhances electron mobility and increases the electroactive surface area, leading to improved sensitivity and lower detection limits^39^. The porous and hydrophilic nature of cellulose promotes efficient electrolyte diffusion, while its modifiable hydroxyl groups allow covalent immobilization of enzymes, antibodies, or aptamers for selective recognition^40^. In addition, coupling with redox mediators (e.g., ferrocene, methylene blue, or Prussian blue) extends the range of measurable targets and enables label-free detection strategies.

Recent designs exploit impedimetric and capacitive transduction, offering non-faradaic, low-power operation suitable for continuous monitoring. The integration of NFC or Bluetooth modules now also allows for wireless data transmission, enabling real-time readouts without external potentiostats^41^. Collectively, all of these advances are transforming cellulose from a passive support into an active optical and electronic interface, capable of coupling biochemical recognition with digital signal processing for next-generation, field-deployable biochemical sensing.

## Design of cellulose-based sensing devices

**Fig. 4:**
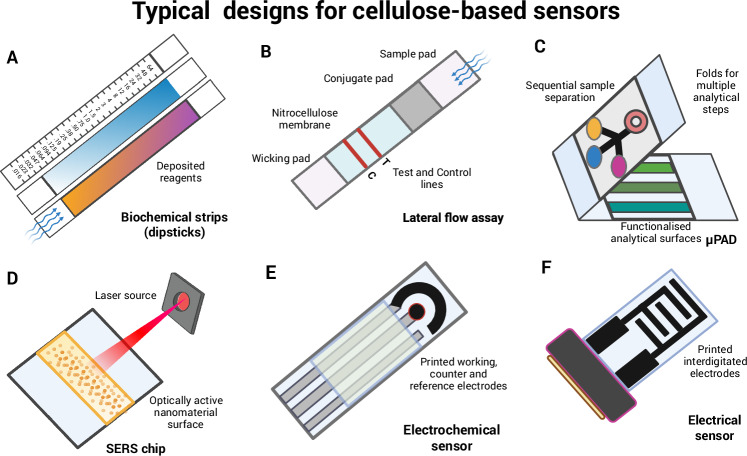
Examples of cellulose-based sensors. **A** The simplest cellulose fabric PoU sensors are biostrips. These are dipped into the analyte solution, where it reacts with a recognition element. This causes a distance-based or intensity-based colourimetric change. These devices may not require a capture element. Introducing different pads gives rise to lateral flow assay formats (**B**), where the analyte travels along or through layered components, interacting with recognition elements in the conjugate pad and capture elements in the nitrocellulose membrane. **C** Folding designs and hydrophobic barriers add further flow complexity, resulting in microfluidic paper-based analytical devices (µPADs). These enable multiplex analyte detection. **D** SERS-based PoU sensor, consisting of a Raman-active paper substrate functionalized with plasmonic nanoparticles and a photodetector, such as a modified smartphone. **E** A three-electrode system for electrochemical sensing and **F** an interdigitated electrode, commonly used for impedimetric and potentiometric measurements. Created with BioRender.com.

The origins of paper-based diagnostic devices trace back to the 1940s, when Martin and Synge introduced paper chromatography to separate and identify plant phenolics^42^. In the 1950s, paper-based tools were adapted to detect glucose in urine, paving the way for the development of modern immunochromatographic devices, such as the home pregnancy test^43^. More recently, the incorporation of microfluidics has enabled multiplexed analyte detection through microfluidic paper-based analytical devices (μPADs) and lab-on-chip systems. In agricultural applications, such cellulose-based point-of-use sensors typically follow certain design architectures: biostrips, LFAs, μPADs, SERS, and electrochemical sensors (Fig. 4).

Biostrips are dry reagent-based platforms that facilitate rapid, colourimetric detection via enzyme-catalyzed reactions on cellulose substrates. Evolving from simple pH litmus strips, modern biostrips incorporate signal amplification strategies using nanomaterials, such as gold nanoparticles or carbon nanotubes, or chromogenic dyes to enhance qualitative or semi-quantitative analysis for environmental and agricultural monitoring^44^.

LFAs employ capillary-driven fluidics to enable single- or multi-analyte detection across a nitrocellulose-based strip, comprising sample, conjugate, reagent, and wicking pads. Molecular recognition is typically mediated by antibodies, aptamers, or CRISPR-based systems, while signal generation is achieved through gold nanoparticles or dyes that yield rapid visual results^45^. Advances in this field include fully cellulose-based LFAs, multiplexed configurations, nanomaterial-enhanced signal amplification, vertical flow formats for improved speed, and smartphone integration for quantitative data acquisition^46,47^.

Compared to LFAs, μPADs can feature more complex geometries, incorporating multiple microfluidic channels to allow for simultaneous detection of several analytes. Flow control is achieved through surface modification with hydrophobic barriers or mechanical incisions, enabling the integration of valves and switches for autonomous fluid manipulation without external pumps^48^. This architectural flexibility makes μPADs a versatile platform with the potential for scalable automation in agricultural diagnostics^49^. μPADs can be readily integrated with portable electronics and spectroscopic techniques to support a range of sensing modalities, including electrochemical, colourimetric, fluorescent, and electrochemiluminescent detection. For example, electrophoretically controlled paper-based electrofluidic systems enable time-resolved, high-throughput assays^50^. These platforms are also compatible with nucleic acid-based screening methods, including CRISPR-based detection, offering a promising route for plant disease diagnostics, though fully cellulose-based implementations remain technically challenging^51^.

For a SERS-based cellulose sensor design, deposited plasmonic nanostructures (typically gold or silver nanoparticle clusters) generate confined electromagnetic fields that boost Raman scattering cross-sections by factors of 10⁶−10⁸ ^52^. When these structures are immobilized on cellulose fibers or formed in situ through nanoparticle aggregation, the resulting nanogaps act as Raman hot spots that enhance sensitivity to the point where single-molecule events can be resolved. The approach becomes even more powerful when combined with microfluidics or LFA architectures, where controlled flow, droplet manipulation, or capillary-driven concentration steps stabilize hot-spot formation and improve analyte localization^53^. These strategies support quantitative and multiplexed detection of pesticides, mycotoxins, heavy metals, and plant metabolites directly from agricultural sample matrices. Although surface-enhanced Raman sensing offers high molecular specificity, its practical deployment on cellulose platforms faces important limitations. Raman spectra are inherently multidimensional, with overlapping vibrational bands that typically require chemometric processing, machine-learning classifiers, or vibrational modeling to extract meaningful information^54^. This computational layer is fundamental to SERS and underpins its discriminative power, yet it becomes a bottleneck in point-of-use scenarios where simplicity, low cost, and rapid interpretation are essential.

Designing cellulose-based electronics typically involves adapting two core architectures: three-electrode electrochemical platforms and interdigitated electrical sensors. In electrochemical formats, cellulose papers or films act as the substrate onto which carbon, metal, or nanocomposite inks are patterned to form working, reference, and counter electrodes^39^. The porous microstructure of cellulose, for instance, enhances electrolyte transport and facilitates interfacial contact between soil extracts or plant exudates and the electrode surface, enabling voltametric or impedimetric detection of nutrients, agrochemicals, or stress biomarkers. In parallel, interdigitated electrode designs leverage the dielectric properties of cellulose to monitor parameters such as moisture, salinity, or ionic strength through capacitance or resistance changes between closely spaced metallic fingers^55^. These devices benefit from cellulose’s wicking ability, mechanical flexibility, and compatibility with low-temperature printing processes, supporting scalable fabrication and direct deployment in soil or on plant tissues. Electrochemical and electrical cellulose-based sensors still encounter issues such as electrode degradation in complex soils and biofouling, which limit their long-term reliability. Performance can also vary due to the anisotropy of cellulose, meaning its fibers absorb water and deform differently along different directions, prompting ongoing efforts to improve stability through surface passivation, nanomaterial reinforcement, and more robust encapsulation. In the following sessions, we will discuss how cellulose-based sensors are being used to monitor soil and plant health, the two major areas involving precision agriculture.

## Soil Monitoring

Soil is a natural, dynamic mixture of minerals, organic matter, water, air, and living organisms that forms the upper layer of the Earth’s surface. Soil supports the growth of plants by providing essential nutrients, anchorage, and a medium for water and gas exchange. Formed over time through the weathering of rocks and decomposition of organic materials, soil plays a vital role in ecosystems, agriculture, and climate regulation^56^.

Defining soil health is inherently complex, as it involves numerous interrelated biological, chemical, and physical processes. For simplicity, it can be broadly categorized into three dimensions: fertility, quality, and security^57^. Soil fertility is directly linked to crop productivity and food output. Soil quality encompasses factors such as water retention and movement, resistance to biotic stress, the effects of agrochemical usage, and contamination. Soil security, on the other hand, refers to the sustainable management of soil resources in a stakeholder-specific and site-adaptive manner. These categories are informed by diverse indicators, including nutrient availability (e.g., nitrogen, phosphorus, sulfur mineralization), biomass production, carbon and nitrogen sequestration capacity, organic matter content, pH, humidity, and temperature^58^. Lehmann et al. recommend that comprehensive soil health indices include at least 20% representation from each category of biological, chemical, and physical indicators, with the relative emphasis adjusted according to specific application goals^56^.

Traditional techniques, such as Fourier transform infrared spectroscopy and ultraviolet-visible methods, can offer broad assessments of soil conditions, but often lack spatial and temporal resolution. In contrast, point-of-use cellulose-based sensors functionalized with responsive materials offer a compelling route toward in-situ, continuous, and analyte-specific soil monitoring^59^. These platforms are uniquely suited to capturing spatial and temporal variations in nutrient availability, soil chemistry, and environmental drivers, information that forms the foundation of seasonally adaptive, data-driven agronomy.

**Fig. 5:**
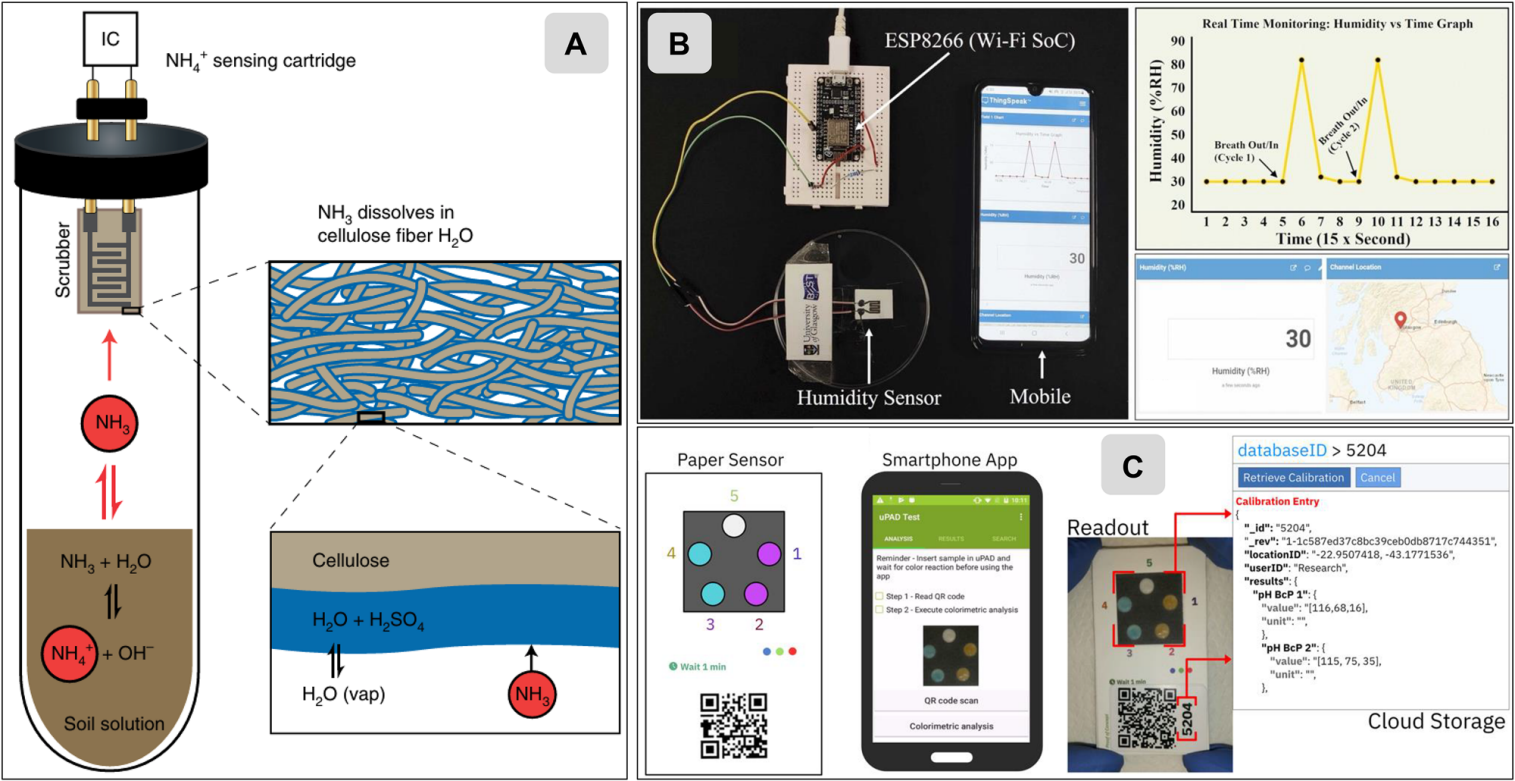
Cellulose-based sensors for soil monitoring. **A** Point-of-use gas-phase ammonium sensor developed by Grell et al.^12^. The disposable chemPEGS element, composed of a cellulose-based scrubber, captures volatilized NH₃ from soil extracts; impedance changes due to acid–base neutralization are analysed electronically, providing a quantitative proxy for soil NH₄⁺ content. **B** Wireless cellulose-paper humidity sensor reported by Beniwal et al.^78^. The resistive graphene–carbon composite enables real-time tracking of soil humidity, with data transmitted to a smartphone interface displaying temporal and geospatial variations. **C** Smartphone-assisted colourimetric paper sensor for soil pH analysis from Ferreira da Silva et al.^70^. The μPAD integrates dual pH indicators, a QR-code reference, and a cloud-linked mobile application for automated image capture, color correction, and geo-tagged pH data storage. All images reproduced and adapted with permission from the original sources under the Creative Commons CC-BY 4.0 license.

Cellulose-based soil sensors target four major domains: fertility, pH, moisture, and biological activity, each requiring different transduction strategies and device designs. In the following sections, we highlight representative examples of cellulose-based technologies that illustrate how paper substrates are being engineered to deliver actionable soil diagnostics and support more nuanced, periodic insights into key agronomic variables (Fig. 5).

### Soil fertility and nutrient profiling

Soil health is fundamentally governed by the availability and balance of 14 essential macro- and micronutrients that are critical for supporting plant growth and sustaining agricultural productivity^60^. Carbon, hydrogen, and oxygen, derived from atmospheric gases and biomass decomposition, are fundamental elements for plant growth. Macronutrients such as nitrogen, phosphorus, and potassium must be absorbed from the soil in ionic forms, including ammonium (NH₄⁺), nitrate (NO₃⁻), nitrite (NO₂⁻), orthophosphate (H₂PO₄⁻, HPO₄²⁻), and potassium ions^61^. These nutrients are essential for photosynthesis, protein synthesis, enzymatic activity, and biomass development. Effective sensing technologies must monitor a wide spectrum of these macro- and micronutrients, with emphasis on nitrogen, phosphorus, and potassium, as they directly affect soil fertility and plant health. For example, nitrogen enrichment through fertilization can enhance protein content and grain yield in crops such as maize and wheat, but excessive application may reduce quality in its processed products, for instance, lowering the glutenin-to-gliadin ratio in bread or the oil and sugar content in rapeseed and sugar beet^62,63^. Detection of nutrients is also closely linked to soil pH, which influences the bioavailability of nutrients and often necessitates context-specific measurement strategies, as discussed below.

Zeitoun et al. present a simple yet thoughtful approach to soil phosphate testing by pre-loading cellulose pads with both the extractant and the color-forming molybdate chemistry^64^. In practice, the user mixes soil with water, adds the pad, and the resulting solution is analysed with a small electrochemical reader. Because the paper itself carries out both extraction and complex formation, the workflow becomes almost effortless. The approach benefits from minimal reagent use, low waste, and a clear electrochemical signal. Still, it depends on harsh extractants and external instrumentation, and it has only been explored in a limited soil set. Moving toward greener reagents, embedding electrodes directly on paper, and expanding to other nutrients would make this concept even more compelling for decentralized testing. Heidari-Bafroui and colleagues took a different angle, where instead of redesigning the chemistry, they enhanced the performance of existing phosphate strips by placing them inside a small infrared lightbox connected to a smartphone^65^. By controlling the lighting and capturing images at a wavelength where the molybdenum-blue complex absorbs strongly, the color changes become more pronounced and far easier to quantify. This strategy is attractive because it builds on familiar, commercially reliable test strips while adding a layer of digital precision. Its limitations lie in the need for an additional piece of hardware and the lack of validation with real soil extracts. Future refinements could shrink the reader into a phone attachment, tune it for other nutrient chemistries, and test the system across more realistic field conditions.

For potassium analysis, Hemavibool and Thongkam proposed a paper-based test that uses patterned cellulose microzones as the final readout surface, although the chemistry behind it still relies on a more traditional extraction and complexation step^66^. After potassium is bound by a crown ether (a polyether cyclical chain molecule) and separated, the remaining color in the water phase is spotted onto the paper and interpreted with a smartphone. The format is inexpensive and uses small reagent volumes, but it remains cumbersome due to the need for organic solvents and multiple handling steps. The indirect nature of the color change may also be less intuitive to non-specialists. Simplifying the workflow by moving more of the extraction onto paper and avoiding hazardous solvents would make the method far more suitable for field use. Giménez-Gómez et al. offer a refreshingly accessible approach by pairing a simple French-press water extraction with a wax-printed paper device capable of measuring nitrate, phosphate, and pH on the same strip^67^. As the extract moves through the patterned channels, colored sections change based on nutrient levels, and a pH indicator shows an instant color change. The appeal here is the combination of true multiplexing with an extraction method anyone can perform, making the platform particularly user-friendly. The trade-off is that water extraction does not match standard agronomic methods, and performance across diverse soils still needs to be mapped. Calibrating the assay to recognize soil-test indices, adding additional nutrients, and integrating app-based interpretation would help this platform mature into a practical field tool.

To detect nitrogen, Grell et al. took an ambitious step beyond conventional soil nutrient tests by pairing a disposable paper-based ammonia sensor with machine-learning models that infer and even forecast overall soil nitrogen status^12^. Their platform uses a simple cellulose strip with a carbon-ink-printed interdigitated electrode functionalized with acid and integrated into a small container. This setup allows volatilized ammonia from a soil extract to neutralize the acid on the paper and generate an impedance change, cleverly sidestepping matrix effects and enabling low-cost ammonium measurements at the point of use. What makes the work distinctive is not only the sensor but the idea of combining this minimal hardware with soil pH, conductivity, and easily accessible weather data to estimate nitrate levels and predict short-term nitrogen dynamics. The strengths are clear: dramatically lower instrumentation requirements, a path toward data-rich fertilization decisions, and a framework that treats soil as a dynamic system rather than a single snapshot. At the same time, the approach depends on training data, has a slow measurement time for the paper sensor, and has only been demonstrated in a limited soil type and controlled conditions. Improving the speed and selectivity of the platform, expanding datasets across diverse soils and fertilizers, and incorporating measurements from real cropping systems would help translate this concept into a robust field-ready nutrient intelligence tool.

### Soil pH

Soil pH functions as a master soil variable shaped by biogeochemical processes involving organic matter decomposition, nutrient mineralization, ammonia volatilization, and microbial activity^68^. Transformations such as nitrification, denitrification, and rhizosphere interactions modulate pH dynamics, strongly influencing plant phenotypes, nutrient uptake, and overall crop yield. Optimal soil pH typically falls between 5.5 and 7.5, although ideal values vary widely across plant species and cropping systems. Environmental drivers, including acid rain, nitrification of ammonium fertilizers, organic matter decomposition, and nutrient uptake, continuously modulate pH in the root zone. During the nutrient uptake, crop roots absorb more cations than anions and release H⁺ ions to maintain charge neutrality, thereby contributing to soil acidification. Conversely, pH elevation stems from irrigation with bicarbonate-rich water, alkaline mineral weathering, and limited leaching under arid soils^69^.

Soil pH was historically assessed using simple color-changing paper strips, but modern approaches now achieve far greater precision through multichromatic indicator papers and advanced electrochemical sensors. A notable recent contribution to cellulose-based soil pH sensing is the mobile colourimetric platform developed by Ferreira da Silva and collaborators, which addresses the persistent challenge of obtaining affordable, high-resolution soil chemistry data directly in the field^70^. The authors fabricated multilayer μPADs by wax-printing hydrophobic barriers onto chromatography paper and depositing bromocresol green and bromocresol purple indicators onto discrete reaction zones, creating a low-cost colourimetric sensor compatible with rapid soil-extract assays. In use, farmers prepare a simple calcium chloride soil extract, apply it to the paper card, and rely on a smartphone application that segments the indicator zones, corrects illumination using onboard color references, and applies a trained machine-learning classifier to categorize soil pH as low, medium, or high. This approach leverages the capillarity and reagent-storage capacity of cellulose while enabling automated, GPS-tagged measurements with minimal user expertise. Its major strengths include compatibility with tropical field conditions, nine-fold gains in spatial resolution compared with standard laboratory compound sampling, and the ability to classify pH with high accuracy when colourimetric development is robust. Key limitations relate to variability introduced by ambient conditions, premature sample evaporation, and the dependency of performance on well-functioning test cards. Future improvements will require more evaporation-resistant paper architectures, expanded libraries of stable colourimetric indicators, and more generalizable machine-learning models that remain reliable across soil types, climates, and smartphone cameras.

Integrating soil pH measurements with heavy metal detection can enable multifunctional sensing platforms to disentangle the underlying causes of soil acidity. A relevant example comes from a study that paired portable X-ray fluorescence with visible–near-infrared spectroscopy to assess farmland soils^71^. The authors demonstrated that combining elemental measurements with pH estimation allowed rapid identification of areas affected by metal contamination and acidity shifts, offering a faster alternative to conventional laboratory analyses. This integrated approach improved spatial coverage and supported on-site decision-making by reducing the need for extensive sampling. The work also highlighted important challenges, as the test performance depended strongly on soil type, moisture, and particle size, which introduced variability in the readings. Some metals could not be accurately detected at low concentrations, and the pH predictions tended to underestimate values for certain soil classes. These limitations indicate that future multifunctional platforms will require improved detection limits, better correction methods for soil heterogeneity, and designs that remain accurate across diverse field conditions.

A recent study by Tohamy introduces a multifunctional cellulose-based pH-sensing platform designed for food safety applications rather than agriculture, yet its material strategy provides insights relevant to cellulose engineering for soil pH sensing^72^. The authors address the challenge of detecting spoilage-related pH shifts and chromium contamination by embedding nitrogen-doped carbon dots, derived from beetroot pigments, into a cellulose sulfate/carboxymethyl cellulose matrix to form a cast film. The composite is produced via solution casting, relying on hydrogen-bonding and electrostatic interactions between the carbon dots and cellulose derivatives, which enhances film polarity, charge-transfer behavior, and pore uniformity. When used as a wrap for tomatoes, the film reports pH changes through betalain-dependent color transitions and detects chromium via rapid pigment oxidation, while also providing antimicrobial activity. Its strengths include the use of biodegradable cellulose chemistry, visible readouts, and the integration of multiple sensing modalities into a single material. Yet the system faces several constraints: pH responsiveness is tied to pigment stability, chromium detection depends on oxidative reactions that may lack selectivity, and performance was demonstrated only in packaged-food environments rather than complex matrices such as soil. Future improvements could focus on replacing food-derived dyes with more stable pH chromophores, validating sensing repeatability under environmental stressors, and adapting the material architecture to interfaces relevant to agricultural substrates, where moisture, ionic strength, and microbial load differ substantially.

### Soil moisture

Soil moisture plays a critical role in regulating biogeochemical processes, nutrient uptake, microbial activity, and plant-pathogen interactions^73^. Effective management of soil moisture is therefore vital, particularly in arid and semi-arid regions, to optimize irrigation practices and ensure food security^74^. Moisture conditions also strongly modulate the pressure of disease occurrence. For instance, *Ralstonia solanacearum* thrives in moisture-saturated soils and causes bacterial wilt in crops such as potato, tomato, and tobacco. In contrast, *Rhizoctonia bataticola* dominates under dry conditions and triggers dry root rot in chickpea. These contrasting pathogen behaviors illustrate how moisture regimes drive distinct disease outcomes^75^. Another example is soybean crops, in which low soil moisture during critical stages such as flowering and seed development can lead to reduced yields and diminished levels of essential nutrients in the next generation of plants^76^. Additionally, moisture levels impact transpiration rates, biomass distribution, and root zone activity, collectively impairing water uptake and increasing plant susceptibility to systemic stress^77^.

Beniwal and colleagues addressed the need for low-cost, flexible, and disposable humidity sensors that can support environmental and soil-monitoring applications without the complexity of fabrication typical of nanomaterial-based platforms^78^. Their approach relies on screen-printing a graphene–carbon ink onto paper substrates to produce resistive humidity sensors that respond to moisture through adsorption-induced changes in film conductivity. The devices are extremely simple to fabricate: single-layer printing on glossy paper produced the most sensitive configuration and can be deployed directly for respiration monitoring or placed on soil surfaces to infer moisture changes via resistance shifts. This straightforward printed architecture offers notable advantages, including low material cost, good mechanical robustness, fast response and recovery, and compatibility with wireless data transmission. Nonetheless, the sensors remain on surface-contact devices rather than true subsurface soil probes, and their performance is heavily influenced by substrate hydrophilicity and environmental drift. Long-term use in agricultural soils may also be limited by the susceptibility of paper to swelling, microbial degradation, and waterlogging. Future improvements could include cellulose-engineered substrates with controlled porosity or hydrophobic coatings to stabilize baselines, integration with biodegradable encapsulation layers to improve durability in wet soils, and adaptation of the printed geometry to allow partial subsurface insertion for more representative soil-moisture readings.

With climate-induced variability increasingly impacting agricultural productivity, effective soil moisture management has become integral for ensuring agronomic stability. For example, in maize cultivation across the USA, soil moisture data is leveraged to predict crop yields by comparing root zone moisture against atmospheric evaporative demand^76^. This highlights the importance of continuous and spatially resolved moisture sensing. While gravimetric drying and time-domain reflectometry remain standard approaches for soil moisture quantification, recent works have introduced cellulose-based capacitive sensors as a low-cost, field-deployable alternative. Capacitive sensors are typically fabricated by printing interdigitated electrodes, using carbon ink, silver ink, or conductive polymer formulations, directly onto cellulose paper or cellulose–polymer laminates. Recently, sputtering defined layers of interdigitated electrodes made of Ti/Ni onto chemically active and derived nanocellulose film have also been used as capacitive sensors that are sensitive to %RH changes as a result of water-induced protonic conduction^79^. The performance of capacitive sensing here depends on the porous nature of the cellulose matrix, which acts as the sensing dielectric: when soil water infiltrates the fibers, the effective permittivity increases, shifting the capacitance measured across the electrodes. Reported detection ranges typically span the full agricultural water content spectrum (5–40% v/v)^80^, with response times of seconds to minutes, depending on fiber density and encapsulation. Because cellulose substrates are inexpensive, biodegradable, and compatible with roll-to-roll manufacturing, they enable large-area, distributed moisture-mapping networks at far lower cost than conventional electronic probes. Coupling these sensors with disease-forecasting or irrigation-decision platforms could help farmers anticipate pathogen outbreaks driven by moisture fluctuations and optimize water use across entire fields^81^.

A recent study by Zaccarin et al. addresses the persistent challenge of deploying low-cost, scalable soil-moisture sensors suited for dense spatial monitoring in precision agriculture by developing a fully biodegradable, wireless capacitive sensor printed on a nanocellulose-reinforced paper substrate^80^. The device is fabricated through screen-printing of conductive silver traces to form interdigitated electrodes coupled to an inductive loop, enabling passive wireless readout through resonant frequency shifts that track changes in soil dielectric properties. In use, these sensors are buried directly in soil and interrogated via an external loop antenna connected to a vector network analyzer, allowing moisture-dependent resonance changes to be monitored over periods exceeding an entire growing season. This approach demonstrates several strengths: an extremely low-cost, biodegradable platform that survives more than 3 months in soil, the ability to detect moisture at depths up to 5 cm, and a wireless architecture that avoids tethered electronics. Yet the technology also faces key limitations. The interrogation range remains short, restricting deployment in deeper soil layers, and sensor performance is impacted by declining quality factors under high-moisture conditions. The use of silver ink constrains full biodegradability, and the need for a laboratory-grade analyzer limits field practicality. Future improvements could include the development of higher-conductivity biodegradable inks, integration with compact or custom interrogation electronics, and strategies to enhance resonance quality factors to extend readout distance. Despite these challenges, this work establishes an important foundation for sustainable, field-deployable cellulose-based moisture sensing within emerging IoT-for-agriculture ecosystems.

### Soil micro- and macrobiome

The soil microbiome is a diverse community of microorganisms, including bacteria, fungi, archaea, protozoa, and viruses, that inhabit the soil and are fundamental to nutrient cycling, decomposition of organic matter, and overall health of the ecosystem. Alongside these microscopic organisms, the soil macrobiome, which includes nematodes, earthworms, insects, mites, and other soil-dwelling macrofauna, also plays a crucial role in maintaining soil structure, promoting aeration and water infiltration, and facilitating microbial activity through bioturbation and organic matter turnover. Together, the micro- and macrobiome form an interconnected biological network that drives soil fertility, pathogen occurrence/suppression through complex trophic and competitive interactions, and plant productivity^82^. While conventional detection techniques, such as nucleic acid amplification and antibody-based assays, provide valuable information to monitor these soil micro and macrobiomes, they remain limited for point-of-use applications due to their complexity and infrastructure demands. Alternatively, monitoring their microbial enzyme activity, such as β-glucosidase, urease, N-acetyl-glucosaminidase, peroxidase, lipase, and phosphatases, serves as a robust, field-relevant proxy for assessing soil biological function and health^83^.

Cellulose-based sensing platforms can be adapted for enzyme-responsive assays to characterize microbial activity and to identify a responsive biome in situ for a given soil condition or a specific plantation site. For example, Cheng et al. developed a cellulose acetate–coated capacitive sensor for real-time assessment of soil microbial activity^84^. The device detects enzymatic degradation of the cellulose acetate layer, mediated by carbon-cycle enzymes such as cellulase, β-glucosidase, and amylase, as a proxy for microbial metabolism. Field trials demonstrated that the sensor could distinguish microbially enriched from sterilized soils, providing a simple and scalable approach to monitor soil health and C-cycle dynamics. The study also highlights how cellulose-based electrochemical platforms can act as indicators of soil biological function and fertility.

The rhizosphere, the soil zone immediately surrounding the plant root, is directly shaped by root growth, its secreted chemicals, and microbial colonization and serves as a particularly rich interface for sensing plant–microbe interactions. Root exudates, which include sugars, amino acids, and signaling molecules such as flavonoids, terpenoids, and strigolactones, shape the microbial communities associated with different plant species^85^. Monitoring changes in those organic compounds can therefore reveal spatiotemporal shifts in microbiome composition, enabling targeted soil interventions and crop breeding strategies. For example, a recent study demonstrated a cellulose-based µPAD designed to monitor glucose exuded by living wheat roots using titanium-dioxide nanotube hydrogels as the sensing element. The device addresses the challenge of non-destructive, spatially resolved sampling of root exudates, traditionally limited by destructive soil extraction or hydroponic artefacts, by using wax-printed paper channels to transport exudates from specific root regions toward the sensor. The µPAD is made by using wax to pattern Whatman filter paper, cutting it with a laser, and adding a hydrogel-based enzymatic glucose sensor at the end of each channel. During use, capillary-driven flow carries root-released glucose to the sensor, where enzymatic oxidation produces a quantifiable blue color proportional to concentration. This enables continuous, multi-day mapping of exudation patterns along individual roots. Strengths include low cost, simple fabrication, spatial resolution, and real-time monitoring. Limitations include long extraction times, modest sensitivity compared to chromatographic methods, and suitability for seedlings rather than mature plants. Opportunities for improvement include integrating electrochemical detection, expanding analyte coverage, and enhancing automation^86^.

Soil nematodes play a pivotal role in nutrient cycling and organic-matter decomposition. Depending on the context, they can serve as indicators of soil health or act as destructive pests that undermine crop productivity^87^. A nematode is a microscopic, worm-like organism (often called a roundworm) that inhabits soil, water, or plant tissues. Many feed on bacteria or fungi, thereby influencing nutrient turnover, while others parasitize plant roots and reduce yield potential^88^. No current cellulose-based sensor has been explicitly developed to detect soil-dwelling nematodes, but model organisms such as *Caenorhabditis elegans* have been used to prototype chitinase-responsive colourimetric assays on paper substrates to monitor its developmental stages, particularly egg hatching. These platforms can also be adapted to evaluate nematocidal agents, such as 5-fluoro-2′-deoxyuridine, with the potential to inform new soil- or crop-selective therapeutic interventions by modulating nematode populations^89^.

## Plant monitoring

Plant growth and productivity are shaped by a variety of biotic and abiotic stressors, which together constrain the effective use of arable land for crop production. These stresses disrupt species-specific physiological, biochemical, morphological, and cellular processes, reducing seed vigor, yield, and nutritional quality while altering the expression of key genetic traits^90^.

Abiotic stress, arising from drought, heat, salinity, or nutrient imbalances, disrupts photosynthesis and mineral uptake, often accelerating plant senescence^91^. Plant senescence refers to the natural ageing process in leaves and other tissues, during which chlorophyll breaks down, photosynthesis declines, and nutrients are remobilized to other functioning parts of the plant. Under abiotic stress, such as drought, heat, or nutrient imbalance, this ageing process is accelerated, leading to earlier yellowing and reduced productivity^92^.

Biotic stress, caused by pathogens such as bacteria, viruses, fungi, oomycetes, nematodes, and herbivorous pests, leads to tissue damage, stunted growth, and reduced yield. As sessile organisms, plants rely on biochemical and proteomic plasticity and innate immune responses to detect and respond to rapid fluctuations in their environment^93,94^. Sensing platforms that target such fluctuations of stress-associated chemical markers and causative pathogens offer a means to detect early physiological changes and support spatially and temporally resolved plant health diagnostics, supporting precision agriculture strategies^94^.

**Fig. 6:**
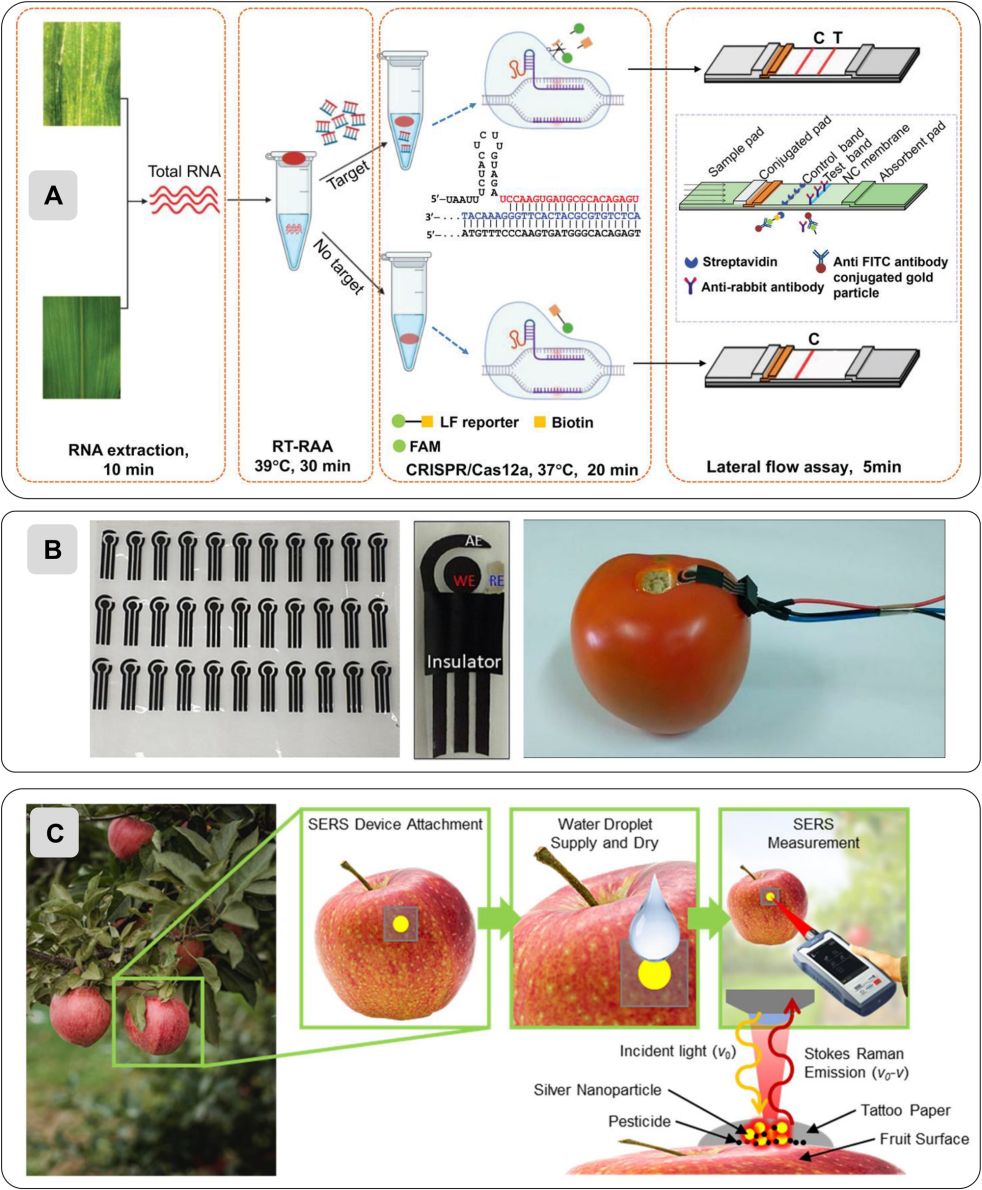
Cellulose-based sensors for plant monitoring. **A** Nitrocellulose-based CRISPR–Cas12a lateral flow assay developed by Lei et al.^102^. for rapid, on-site detection of maize chlorotic mottle virus. The nitrocellulose membrane integrates isothermal amplification and Cas12a cleavage in a portable, lyophilized format, enabling colourimetric readout without laboratory equipment. **B** Sustainable cellulose-based wearable sensor for pesticide detection reported by Teixeira et al.^121^. The flexible electrochemical device, fabricated on cellulose acetate and carbon-based inks, conforms to fruits for in-situ measurement of pesticide residues under field conditions. **C** Tattoo paper-based SERS substrate designed by Mandrekar et al.^122^. The inkjet-printed Ag-nanoparticle film allows direct adhesion to fruit surfaces, providing non-destructive, ultrasensitive pesticide residue analysis via surface-enhanced Raman spectroscopy. All images reproduced and adapted with permission from the original sources under the Creative Commons CC-BY 4.0 license.

In addition to chemical and physical indicators, plant electrical signals, including local electrical potential, action potentials, variation potentials, and systemic potentials, are increasingly recognized as significant physiological signals that encode a plant’s rapid responses to environmental stimuli such as wounding, temperature change, and water status. These bioelectrical signals propagate within the plant vascular and cellular networks and can reveal stress responses, coordination of physiological processes, and systemic communication across organs. Such signals are measurable as changes in membrane potential or extracellular surface potentials and have been studied with electrophysiological tools in model plants (e.g., action potentials in Venus flytrap and Mimosa) and broader taxa^95^. While the majority of cellulose-based sensors to date have focused on chemical and environmental analytes, integrating electrophysiological measurement with biodegradable or flexible substrates (for example, bacterial cellulose electrodes interfaced to plant surfaces) suggests a future opportunity to develop cellulose-enabled plant electrical monitoring platforms that could complement chemical sensing and support real-time assessment of plant stress and health^96^. Figure 6 shows examples of different cellulose-based sensors for plant monitoring.

### Biotic stress

Detecting biotic stress typically involves identifying molecular signatures associated with pathogen invasion. These targets include pathogen-derived nucleic acids, virulence-associated proteins, secreted enzymes such as proteases, and other extracellular markers produced during host–pathogen interactions^97^. Their detection is commonly transduced through colourimetric changes or electrochemical readouts, enabling rapid on-site assessment. Plant tissues, such as leaf, stem, or root extracts, can often be applied directly to these platforms with minimal preparation, although nucleic-acid-based assays may benefit from simple extraction steps.

To support integration with paper-based formats, including lateral-flow devices and bioactive dipsticks, several commercial kits are available that provide streamlined extraction workflows compatible with low-resource field use. Examples include the vial-based DNA extraction kit (OptiGene Ltd, UK), Spotcheck extraction buffer (Neogen, USA), plastic extraction bags (Agdia Inc., USA), and nylon mesh-based extraction bags (BIOREBA, Switzerland). These kits facilitate the efficient release of nucleic acids from rigid plant cell walls, producing lysates suitable for downstream detection with paper-based diagnostic tools^98^.

Recent advances have pushed cellulose far beyond simple colourimetric strips, transforming it into a multifunctional scaffold for nucleic-acid amplification, enzyme activity monitoring, immunochemical recognition, and CRISPR-enabled detection. Khaliliazar et al. introduced a multilayer paper device that brings nucleic acid amplification and electrochemical readout onto a compact, cellulose-based platform, an appealing direction for early plant pathogen detection^99^. Their system uses stacked wax-patterned paper, glass-fiber pads preloaded with amplification reagents, and gold thread electrodes that guide hybridization and signal transduction. Once nucleic acids are amplified isothermally, reporter strands form an electroactive complex on the immobilized probes, allowing sensitive detection with a small portable potentiostat. The format is modular, inexpensive, and compatible with freeze-dried reagents, highlighting its potential for plant-disease diagnostics where lab infrastructure is limited. Still, controlled heating, multilayer assembly, and reliance on external readers limit true field portability. Improvements could simplify the mechanical sequencing steps, integrate low-cost electronics, and tailor the assay to major plant viruses and fungi. This work sets the stage for more complex nucleic-acid paper systems used later in this section.

Building on the idea of embedding biochemical reactions directly into cellulose, Choi et al. develop a wax-printed, origami-style paper device that detects pectin-degrading enzymes associated with *Allium* white rot^100^. The design hosts pre-loaded ruthenium red complexes that undergo a distinct color change when fungal polygalacturonase cleaves pectin, offering a functional, enzyme-level view of plant stress. The device is simple to produce, requires only small sample volumes, and provides an intuitive visual readout that can be enhanced with smartphone color extraction. Its strength lies in capturing early biochemical changes in infected tissue, complementing the genetic specificity of nucleic-acid platforms like Khaliliazar’s^99^. Yet, performance remains sensitive to temperature, pH, and incubation time, and targets only a single enzyme that may vary across strains. Greater stability, integrated tissue preparation, and multiplexing with other cell-wall-degrading enzymes would broaden its utility, but it illustrates how cellulose can host both genetic and enzymatic stress markers.

Miranda et al. extend cellulose-based sensing into immunodiagnostics, adapting nitrocellulose lateral-flow formats for early detection of Asian soybean rust^101^. Their strip uses immobilized fungal antigens and fluorescently labeled antibodies to visualize infection with higher sensitivity than conventional gold nanoparticles, yet retains the familiar simplicity of LFA workflows. When the leaf extract is applied directly, the sample wicks through the membrane, and diagnostic fluorescence appears as a clear band under a handheld lamp. This work bridges the molecular specificity seen in nucleic-acid devices and the simplicity of enzymatic assays, offering a practical compromise for farmers who need rapid, actionable results. The trade-off here is dependence on fluorescence readers and potential cold-chain storage requirements for antibody-nanoparticle conjugates. Improving reagent stability, introducing brighter colourimetric labels, and multiplexing several foliar pathogens would enhance field applicability. This method connects well to the expanding trend of turning cellulose membranes into portable immunochemical platforms.

Moving further toward highly specific cellulose diagnostics by coupling isothermal amplification, Lei and collaborators developed a nitrocellulose lateral-flow assay for maize chlorotic mottle virus^102^. After a crude alkaline-PEG extraction, viral RNA undergoes rapid RAA amplification and activates Cas12a, which cleaves a reporter detectable on a cellulose strip. The one-tube chemistry, compatibility with lyophilization, and intuitive strip-based output make this format attractive for decentralized screening in crop fields. Limitations include multiple timed steps, a narrow optimal temperature range, and a focus on a single viral agent. Future developments could integrate internal controls, broaden detection to co-infecting viruses, and embed the chemistry into enclosed reaction cassettes. Within the broader narrative, this work highlights the sophistication possible when cellulose hosts modern genome-editing enzymes. Together, these works illustrate a growing convergence between paper microfluidics, molecular biology, and low-cost materials engineering, showing how cellulose is evolving into a versatile platform capable of supporting both sophisticated molecular assays and accessible, farmer-friendly diagnostics.

### Abiotic stress

Plants exhibit characteristic chemical signatures in response to abiotic stresses. These signatures include: (i) shifts in inorganic ion composition and pH, such as altered Na⁺/K⁺ ratios and Na⁺ accumulation under salinity stress, transient cytosolic Ca²⁺ elevations under herbivore or insect attack, cold stress or mechanical damage, and changes in nitrate and ammonium levels during nutrient deficiencies^103^;(ii) variations in gaseous and water fluxes, for example carbon dioxide and water vapor that report stomatal conductance and drought status and reflect plant–atmosphere water balance^104^; (iii) reactive oxygen and nitrogen species, including hydrogen peroxide, singlet oxygen, superoxide, and nitric oxide, which are rapidly produced during heat, drought, high light, salinity, cold, or pathogenic stress^105^; and (iv) phytohormones such as auxins, abscisic acid, melatonin, salicylic acid, jasmonates and ethylene, which regulate developmental and defense responses^106^.

Conventional methods for detecting the above-mentioned low-molecular-weight compounds (typically below 900 Da) often rely on complex laboratory-based instrumentation, including liquid chromatography coupled with mass spectrometry (LC-MS) and gas chromatography-mass spectrometry (GC-MS) systems^107^. These techniques involve chromatographic separation of analytes followed by sensitive mass-selective detection and require skilled operation, solvent systems, and controlled laboratory environments. In contrast, cellulose-based platforms provide minimally invasive routes for monitoring plant stress metabolites directly in the field, but they vary in maturity, robustness, and analytical performance. For instance, a pyrite-ferrous sulfide sensor for methyl jasmonate detection demonstrates the use of a conductive, defect-rich nanomaterial grown directly on cellulose, achieving sub-micromolar to millimolar detection with smartphone readout^108^. Technically, its main advantages are the low-cost fabrication (~$0.02 per strip), strong electrocatalytic behavior, and Bluetooth-enabled connectivity; yet, it has not yet been evaluated using real leaf extracts, and the high operating potentials (≈1.2–1.3 V) and NaOH electrolyte pose challenges for field safety and matrix tolerance. The concept is promising, but its practical deployment will depend on stability in complex plant sap and the development of a safer point-of-use sampling protocol.

Similarly, recent paper-based systems for proline (a key drought, salinity, and UV stress biomarker) provide strong demonstrations of how cellulose architecture influences analytical sensitivity. The multilayer origami μPAD by Choi et al. incorporates an enclosed mixing channel that greatly enhances reagent–analyte interaction, improving the limit of detection (23 μM) by ~29-fold over their earlier design^109^. The clear advantage is sensitivity and analytical rigor (e.g., validation against spectrophotometry), but the need for 150 °C heating for 9 min increases power consumption and limits true field portability. The device is well-suited for controlled environments or greenhouse monitoring, but remains too energy-intensive for remote agriculture unless paired with alternative heating strategies (solar, chemical, or NFC). Park and co-workers addressed several of these limitations by introducing a semi-enclosed single-layer μPAD with an isatin–chitosan conjugate, reducing the required temperature to 120 °C and the reaction time to only 2 min while maintaining a similar LOD (~23 μM)^110^. The leak-proof geometry and improved reagent immobilization produce stronger, more uniform colourimetric signals, and the smartphone imaging box standardizes readout. The trade-off is specificity: although isatin chemistry is more selective for proline than ninhydrin, some amino-acid-rich matrices may still require controlled extraction conditions. Nonetheless, this design represents a meaningful step toward deployable, low-power plant-stress diagnostics.

With nanocellulose becoming widely researched as a multifunctional cellulose source, recent studies have used bacterial nanocellulose to develop a strong SERS substrate for measuring such small molecules. For instance, Xu and co-workers use bacterial nanocellulose fibers as a porous, biodegradable scaffold to hold densely packed silver nanoparticles, creating a composite flexible SERS substrate capable of detecting plant exogenous salicylic acid down to 10⁻⁷ M (1.4 μg L⁻¹) (salicyclic acid here is used as a plant protectant and growth promoter) while retaining performance over 90 days of storage^111^. By combining nanocellulose’s structural integrity with plasmonic enhancement, the platform delivers a potentially robust, low-cost biochemical sensing methodology.

### Wearable sensors for plants

**Fig. 7:**
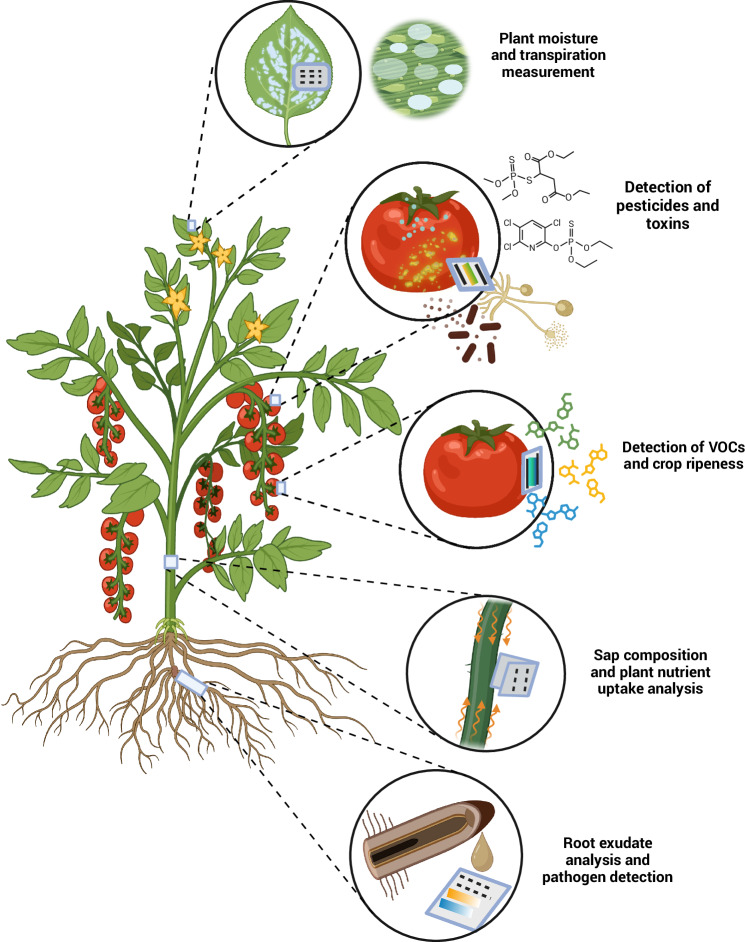
Wearable cellulose-based sensors for real-time plant monitoring. Representative examples of cellulose-based wearable devices applied to distinct parts of plants for continuous and non-destructive monitoring of physiological and environmental parameters. Such platforms exploit the flexibility, biocompatibility, and biodegradability of cellulose to enable conformal contact with biological tissues. Applications include leaf-mounted sensors for detecting chlorophyll content, transpiration rate, or surface temperature; fruit-attached sensors for monitoring sugar accumulation, volatile organic compounds, and ripening-associated metabolites; and stem- or branch-integrated sensors capable of tracking sap flow, turgor pressure, or phytohormone levels. Root-embedded or soil-contact sensors can measure ion concentration, nutrient availability, and moisture dynamics directly within the rhizosphere. Collectively, these wearable cellulose-based devices provide a sustainable framework for precision agriculture, bridging plant physiology, materials science, and data-driven crop management. Created with BioRender.com.

Originally developed for monitoring human health, wearable technologies are now being adapted to measure plant physiological and stress responses (Fig. 7). These devices work by making close contact with leaves, stems, or fruits, where they turn small biochemical or biophysical changes into electrical or optical signals. Typical platforms use flexible electrodes or conductive polymers to track variations in sap flow, transpiration, surface humidity, or ionic leakage, while other designs integrate microfluidic channels or colourimetric films to sense metabolites released at the leaf surface. By continuously converting these plant-generated cues into quantifiable outputs, wearable sensors provide real-time insight into stress progression and environmental influence in plant systems.

Cellulose materials offer a natural advantage for plant-wearable sensors because their porosity, flexibility, and moisture-handling properties closely match the mechanical and physiological characteristics of plant tissues. This compatibility allows cellulose-based devices to conform to curved or delicate surfaces, such as leaves and stems, without disrupting growth or causing mechanical stress^112^. Building on these properties, cellulose fabrics provide a lightweight and biocompatible scaffold for integrating electrochemical or optical sensing elements, while their inherent electrofluidic behavior supports efficient transport of water, ions, and metabolites to the sensing interface. As a result, cellulose wearables can be gently laminated onto plant organs to monitor processes such as transpiration, nutrient flux, gas exchange (including carbon dioxide and volatile organic compounds), and temperature in a minimally invasive manner, creating a seamless interface between soft plant tissues and functional electronics^113^. Such real-time measurements support the assessment of plant health and its associated chemical ecology.

An example is a colourimetric paper-based sensor array that detects *Phytophthora infestans* by sensing volatile organic compounds and phytohormones released from the leaf headspace, using gold nanorods and dyes^114^. This platform demonstrated a 95% detection accuracy and stability of 2 days. Similarly, ripeness of fruits, such as bananas, can be monitored by detecting ethylene, a key ripening hormone, using cellulose-based chemiresistive sensors that incorporate single-wall carbon nanotube - palladium nanoparticle composites^115^. For non-climacteric crops (fruits that do not continue to ripen after harvest) like mangoes, respiration markers such as carbon dioxide and oxygen can be tracked using methylcellulose-based colourimetric films in sealed containers, enabling interference-free sensing^116^. These wearables provide actionable insights for optimizing harvest timing, post-harvest storage, and supply chain logistics, thereby improving crop quality and reducing waste.

To detect crop ripeness, a nanocellulose-enabled optical sensor was developed by Pirsa and Chavoshizadeh, consisting of an ethylene gas sensor based on a nanofiber bacterial cellulose film doped with potassium permanganate^117^. The high porosity and moisture permeability of bacterial cellulose facilitated gas diffusion and supported a colourimetric sensing mechanism, in which ethylene-induced reduction of permanganate produced quantifiable changes in film color, clarity, and absorbance. The sensor was successfully applied to monitor ethylene release in banana packaging, enabling estimation of storage time and temperature under realistic post-harvest conditions. While the approach demonstrates the suitability of nanocellulose films as optical sensing substrates for agri-food monitoring, the sensing response relies on a consumptive chemical reaction and external dopants, highlighting ongoing trade-offs between simplicity, reusability, and long-term stability in optical nanocellulose sensors.

Beyond classical indicators, plant wearables also enable access to new physiological data streams, including dynamic nutrient fluxes, water levels, transpiration rates, photosynthetic activity, and wetness of leaves. Monitoring these parameters continuously reveals microclimatic shifts, symptoms of disease, and stress responses, which could inform irrigation, fertilization, crop breeding, and climate-resilient strategies^118^. Several proof-of-concept systems illustrate these potentials. For example, Dey and collaborators developed a paper-based chipless radio-frequency sensor that demonstrates how cellulose substrates can support non-contact monitoring of plant hydration^119^. The chipless RFID design printed with conductive silver ink functions as a passive microwave resonator whose frequency response shifts with changes in leaf wetness. This approach offers a low-cost, fully printable alternative to commercial dielectric leaf-wetness probes and is attractive for distributed sensing in greenhouses. Its main strengths are simplicity, printability, and wireless interrogation. Yet, the sensor requires leaf-specific calibration because resonance shifts vary with morphology and water content, and signal quality deteriorates in leaves with high dielectric loss. Improving robustness will require strategies that correct sample-to-sample variability, potentially through machine-learning-assisted calibration, more conductive inks, or hybrid designs less sensitive to leaf heterogeneity.

A complementary strategy used pyrolyzed graphitic paper electrodes laminated directly onto leaf surfaces to track water loss through impedance changes. Tinoco et al. tackled the long-standing limitation of pyrolyzed paper, its high resistivity and fragility, by annealing cellulose paper at 800–1000 °C in isopropanol vapor, producing a thin (~15 nm), highly graphitic nanolayer that dramatically reduced resistivity and improved mechanical robustness^120^. The interdigitated electrodes were patterned into an interdigitated using a knife plotter and attached to soybean leaves using transparent medical tape, enabling real-time, non-destructive monitoring of water loss by tracking impedance changes across the leaf tissues during desiccation. This graphitized-paper architecture offers clear advantages: scalable fabrication, benign chemistry, strong charge-transfer kinetics, and excellent sensitivity to changes in leaf water content, while preserving biocompatibility, as the electrodes did not disrupt stomatal structures even after 4 weeks on the leaf. Yet, the devices still face constraints, including hydrophobic surfaces, susceptibility to environmental variation, and uncertain long-term adhesion during outdoor use. Further improvements may require hydrophilic or amphiphilic surface coatings, optimized encapsulation for field deployment, and wireless integration to convert these proof-of-concept wearable electrodes into practical decision-support tools for precision irrigation.

Plant wearables can also be used for the assessment of crop safety, monitoring pesticide residues directly on the surface of crops. For example, Teixeira and colleagues developed a plant-wearable electrochemical sensor to address the persistent challenge of monitoring pesticide residues directly on crop surfaces, where conventional laboratory assays remain impractical for rapid field deployment^121^. Their approach replaces petrochemical polymer substrates with cellulose acetate films produced by solvent casting, onto which carbon and silver inks are screen-printed to form a flexible three-electrode system. Once attached to leaves or fruit skins, the device operates through differential-pulse or square-wave voltammetry to quantify carbendazim and paraquat, relying on the redox activity of each pesticide rather than on biological recognition. This design offers meaningful advantages: the substrate is biodegradable, mechanically resilient during bending, and compatible with direct on-surface measurements without sample extraction. The sensor also demonstrates good selectivity across interfering compounds and maintains performance across repeated flexions, supporting its potential for in-field use. Yet the platform has clear limitations. The electrochemical response diminishes after multiple measurements due to biofouling; the method still requires manual application of buffer onto the leaf, and the reliance on organic solvents during film casting reduces the overall sustainability claim. Signal shifts across pH values also indicate that broader environmental robustness will require further optimization. Future improvements could focus on solvent-free film fabrication, anti-fouling surface treatments, single-use low-cost cartridges to avoid drift, and integration with microfluidic handling layers that autonomously deliver electrolytes. Such refinements would help consolidate cellulose acetate as a practical substrate for on-plant chemical sensing in real agricultural settings.

A study by Mandrekar and colleagues introduced a wearable tattoo-paper SERS platform designed to simplify pesticide detection directly on fruit surfaces^122^. The technology aims to bypass laborious sampling and extraction steps that limit current field-deployable residue testing. Their devices are produced through low-temperature inkjet printing of silver nanoparticle patterns onto commercial tattoo paper, followed by prolonged UV-mediated carbon-residue decomposition to improve spectral clarity. In use, the adhesive cellulose-based tattoo film is wetted and transferred onto curved surfaces, bringing pesticide molecules into intimate contact with plasmonic nanoparticles; Raman scattering is then recorded through the transparent carrier layer. The approach offers clear strengths: it is inexpensive, mechanically conformable, and enables direct, in situ sensing without swabbing or solvent extraction. Yet several weaknesses remain. The fabrication requires 60 h (about 5 days) of UV treatment to remove carbon residues, limiting scalability, and the reliance on nanoparticle clustering leads to variability in hotspot density and signal reproducibility. In real produce, surface heterogeneity and wax layers may further complicate analyte transfer to the SERS layer. Future improvements could focus on faster photochemical cleaning, engineered nanostructures with controlled gap spacing, and more robust quantification strategies that correct surface-to-surface variability. Recent reports are pushing SERS toward direct, on-surface pesticide screening by turning nanocellulose into flexible “wipe-and-read” plasmonic patches that conform to rough crop surfaces. For instance, a monodisperse Ag nanoparticle–decorated bacterial nanocellulose enabled paste-and-read detection of the fungicide thiram on irregular fruit surfaces down to ~10⁻⁸ M^123^. while a 3D dialdehyde/-oxidized cellulose nanofibril film that immobilizes Ag nanoparticles to create stable, high-density hot spots for detecting thiram and thiabendazole to 10⁻⁹ M directly on apple surfaces, with >60-day shelf stability.

## Challenges and outlook

Cellulose-based sensors occupy a unique position at the interface of sustainability, accessibility, and decentralized analytics. Their growing adoption in agricultural sensing is driven by a combination of material abundance, biodegradability, passive microfluidics, chemical versatility, and compatibility with digital readout tools. At the same time, translating these advantages into robust, scalable field technologies requires addressing several interrelated challenges in materials engineering, device design, and data integration.

Environmental sensitivity and mechanical stability remain primary limitations of cellulose-based platforms. The hygroscopic nature of cellulose leads to swelling, deformation, and baseline drift under humid or water-saturated conditions, which can distort electrochemical signals and shorten device lifetime. To address this, emerging approaches focus on cellulose–nanocomposite architectures, surface passivation layers, and permeable protective coatings that preserve capillarity while limiting excessive moisture uptake. Reinforcement with nanocellulose, polymer blends, or elastomeric encapsulation has also shown promise in improving mechanical robustness without sacrificing biodegradability.

Electrical and structural heterogeneity presents a second challenge, as natural variations in fiber density, orientation, and dielectric properties can compromise reproducibility and hinder integration with electronic readouts. This limitation is being addressed through the use of regenerated and engineered cellulose substrates (e.g., lyocell, bacterial cellulose, nanocellulose papers), which offer greater uniformity and tunable microstructure. In parallel, additive manufacturing methods such as screen printing and inkjet deposition of conductive inks enable better control over electrode geometry and electrical pathways, improving device-to-device consistency.

While cellulose substrates offer clear sustainability advantages, the environmental footprint of cellulose-based sensors must be evaluated at the level of the complete device rather than the substrate alone. Conductive components such as silver or gold inks, metal nanowires, carbon nanomaterials, and redox-active labels can contribute disproportionately to material cost, resource extraction, and end-of-life impact. For example, silver nanowires and nanoparticle inks, though effective for low-resistance printed electrodes, raise concerns related to metal scarcity, energy-intensive processing, and potential ecotoxicity if released into soil environments. To address these limitations, there is a growing interest in alternative conductive strategies, including carbon-based inks, biodegradable or transient conductors, conductive polymers, and hybrid architectures that minimize metal loading or confine non-biodegradable components to reusable electronic modules. A holistic assessment of sustainability, incorporating material sourcing, fabrication energy, operational lifetime, and disposal pathways, is therefore essential to ensure that cellulose-based sensing platforms deliver genuine environmental benefits at scale.

Limited long-term stability and reusability constrain most cellulose-based sensors to single-use applications, restricting their utility for continuous or seasonal monitoring. Recent work is therefore shifting towards hybrid designs that decouple the biodegradable sensing interface from reusable electronic components. Battery-free architectures, including NFC- and RFID-enabled electrochemical or capacitive sensors, allow disposable cellulose elements to be paired with reusable readers, extending functional lifetime while preserving sustainability.

Optical and signal variability, particularly in colourimetric and fluorescent formats, represents another barrier to quantitative reliability. Ambient lighting conditions, camera hardware differences, and user-dependent imaging introduce significant variability in signal interpretation. This challenge is increasingly mitigated through smartphone-assisted readout combined with computer vision, education in color calibration standards, and machine-learning-based classification models. These approaches transform simple visual signals into reproducible digital outputs and enable aggregation of measurements across users and locations.

Beyond academic research, industrial interest in cellulose-based and cellulose-enabled sensing technologies is beginning to emerge across both materials manufacturing and precision-agriculture ecosystems. For example, companies such as Borregaard, CelluForce, FiberLean Technologies, and Stora Enso are advancing nanocellulose and functional cellulose materials that are increasingly explored as substrates for printed electronics and sensing interfaces. In parallel, agritech companies such as CropX, Arable, and METER Group illustrate the growing commercial demand for scalable, field-deployable soil and plant monitoring systems, even though most current products rely on conventional polymers or rigid electronics. Translating cellulose-based sensors into large-scale agricultural deployment remains constrained by several bottlenecks, including variability in cellulose microstructure, environmental sensitivity to moisture and mechanical stress, limited long-term stability in soil environments, and the disproportionate contribution of non-cellulosic components (e.g., conductive inks, nanomaterials, and electronics) to overall system cost and sustainability. Addressing these challenges will require coordinated advances in cellulose materials engineering, scalable manufacturing and standardization, and system-level integration with power, communication, and data infrastructures, rather than further diversification of device formats alone.

Looking ahead, the evolution of cellulose-based sensing technologies is likely to follow a staged trajectory. In the near term (2–5 years), progress will focus on multiplexing μPADs and LFAs with smartphone quantification, NFC-enabled electrochemical sensors for soil and plant monitoring, and improved biodegradable printed electrodes with enhanced stability. In the midterm (5–10 years), distributed networks of battery-free sensors, integration with irrigation and fertilization control systems, and hybrid cellulose–electronic devices capable of wireless communication are expected to emerge. In the longer term (>10 years), cellulose-based platforms may mature into adaptive sensing fabrics embedded within closed-loop agricultural systems, enabling continuous monitoring of crop stress, environmental change, and resource use while interfacing seamlessly with climate, carbon, and decision-support frameworks.

Together, these developments position cellulose not merely as a passive substrate, but as a foundational material for decentralized, intelligent, and circular sensing infrastructures. By explicitly coupling material-level challenges with targeted engineering and data-driven solutions, cellulose-based sensors can bridge laboratory-grade analytics with the dynamic constraints of real-world agriculture, supporting more resilient, data-informed, and equitable precision-farming practices.
